# Chronic stress inhibits testosterone synthesis in Leydig cells through mitochondrial damage via Atp5a1

**DOI:** 10.1111/jcmm.17085

**Published:** 2021-12-10

**Authors:** Xiaofan Xiong, Qiuhua Wu, Lingyu Zhang, Shanfeng Gao, Rufeng Li, Lin Han, Meiyang Fan, Miaomiao Wang, Liying Liu, Xiaofei Wang, Chunli Zhang, Yanlong Xin, Zongfang Li, Chen Huang, Juan Yang

**Affiliations:** ^1^ Western China Science and Technology Innovation Port in Precision Medicine Institute The Second Affiliated Hospital of Xi'an Jiaotong University Xi'an China; ^2^ Department of Cell Biology and Genetics, School of Basic Medical Sciences Xi’an Jiaotong University Health Science Center Xi’an China; ^3^ Key Laboratory of Environment and Genes Related to Diseases Ministry of Education of China, Xi’an Jiaotong University Xi’an China; ^4^ Center of Medical Genetics Northwest Women’s and Children’s Hospital Xi’an China

**Keywords:** Atp5a1, Leydig cells, mitochondrial dysfunction, testosterone synthesis

## Abstract

Stress is one of the leading causes of male infertility, but its exact function in testosterone synthesis has scarcely been reported. We found that adult male rats show a decrease in bodyweight, genital index and serum testosterone level after continual chronic stress for 21 days. Two‐dimensional gel electrophoresis (2‐DE) and MALDI‐TOF‐MS analysis identified 10 differentially expressed proteins in stressed rats compared with controls. A strong protein interaction network was found to be centred on Atp5a1 among these proteins. Atp5a1 expression significantly decreased in Leydig cells after chronic stress. Transfection of Atp5a1 siRNAs decreased StAR, CYP11A1, and 17β‐HSD expression by damaging the structure of mitochondria in TM3 cells. This study confirmed that chronic stress plays an important role in testosterone synthesis by regulating Atp5a1 expression in Leydig cells.

## 1 INTRODUCTION

Male infertility not only severely affects families but also places a heavy burden on society. In addition to genetic and environmental factors, psychological trauma is an important factor affecting male reproduction.^1^, ^2^ Male infertility is mediated by multiple mechanisms, including structural damage to the testis blood–testis barrier, inflammation and dysfunction of Sertoli and Leydig cells. Perturbation of the hypothalamic–pituitary–adrenal axis and hypothalamic–pituitary–testicular axis, as well as oxidative stress interference with signalling pathways or epigenetic gene regulation, is involved in the regulation of reproductive function.^3^ Under stress conditions, adrenocorticotropic hormone is secreted into the blood after stimulation with corticotropin‐releasing hormone in the hypothalamus; follicle‐stimulating hormone and luteinizing hormone are inhibited by blocking of gonadotropin‐releasing hormone, thereby inhibiting the secretion of testosterone, decreasing sperm number and vitality and damaging male reproductive function.^4^

Previous scientific evidence has suggested that psychological stress affects spermatogenesis, mainly through the regulation of secretion of nerve and endocrine hormones. Testosterone is an important hormone that maintains male reproductive function, and 95% of the testosterone in mammals is secreted by Leydig cells in the testes. StAR in the mitochondria of Leydig cells regulates the transportation of cholesterol from the outer to the inner mitochondrial membrane; the cholesterol is then transferred to CYP17A1 and converted to pregnenolone. A portion of pregnenolone passes through 3β‐HSD in the endoplasmic reticulum of the sliding surface. The remainder is directly converted into dehydroepiandrosterone by CYP17A1, then metabolized into rostenedione by 17β‐HSD and finally directly converted into testosterone.^5^ The steroid hormone synthase and proteins including StAR, CYP17A1, 3β‐HSD and 17β‐HSD, which are required for testosterone synthesis, are mainly distributed in the mitochondria. In recent years, mitochondria have increasingly been recognized for their important roles in fertility. Testosterone has been reported to regulate the expression of mitochondrial genes and alleviate oxidative damage,^6^ and mitochondrial antioxidants protect steroidogenesis, on the basis of the expression of testosterone and its related steroid synthase.^7^ Lipoprotein metabolism induced by obesity disrupts the electron transport chain and ultimately leads to reduced mitochondrial membranes and inhibits testosterone biosynthesis in Leydig cells.^8^ In addition, acetamiprid drugs have a similar effect.^9^ Thus, mitochondrial dysfunction has been speculated to be closely associated with testosterone synthesis and male reproduction. However, the regulation of cholesterol transport in Leydig cells under stress requires further study. This study aimed to identify changes in gene expression patterns in the testis induced by chronic stress. We used proteomics techniques and then screened for differential indicators involved in male fertility to explore whether stress induces mitochondrial damage and regulates abnormal testosterone synthesis.

Proteomics technology has been widely used in research on a variety of clinical diseases, and is a mature technical method for studying the testis proteome in mammals. Proteomic studies have shown that human sperm contains 20 differentially expressed proteins, thus providing a basis for identifying infertility‐related proteins through proteomics technology.^10^ In addition, 2‐DE and mass spectrometry analysis have identified the roles of prostatic acid phosphatase and prostate‐specific antigen in sperm damage and normal conditions in humans,^11^ as well as other proteins involved in human reproduction.^12^

**FIGURE 1:**
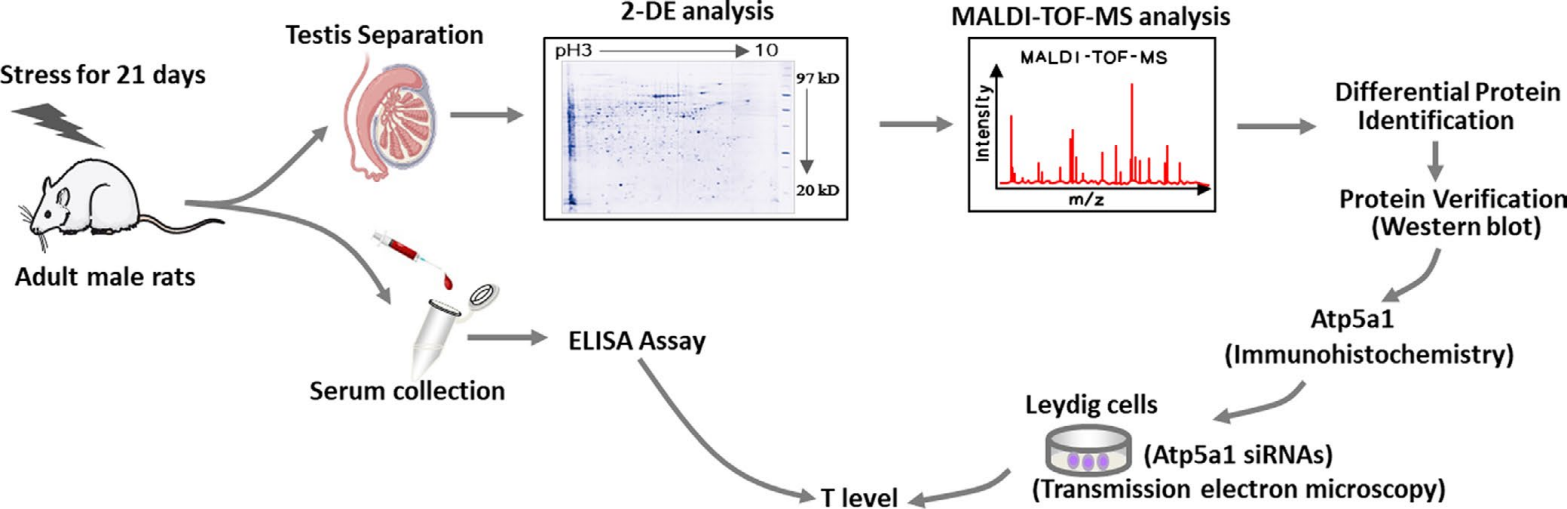
All experimental procedures performed in this study

In summary, the potential mechanisms of molecules involved in male reproductive dysfunction can be explored through testicular proteomics analysis. Herein, 2‐DE combined with MALDI‐TOF‐MS analysis was used to identify changes in the testicular proteome under chronic stress, to reveal the protein expression associated with testosterone synthesis (Figure 1). Subsequently, TM3 cells were used to determine whether the differential indicators might be involved in the testosterone synthesis pathway. This study provides a reliable theoretical basis on the effects of human psychological pressure on male reproductive function.

## 2 METHODS AND MATERIALS

### 2.1 Animals and model preparation

Forty‐eight healthy adult male SD rats (280 ± 20 g) were purchased from the Animal Center of Xi'an Jiaotong University (Medical Experimental Animal Centre of Shaanxi Province, China), and randomly divided into stressed and control groups (*n* = 24 per group, four rats per cage) after a 7‐day adaptation period. The rats were kept at a constant temperature of 22 ± 2°C and a humidity of 50%, and were given free access to drinking water and food. All experiments were performed in accordance with the relevant guidelines and regulations, and all animal procedures were approved by the Animal Ethics Committee of Xi'an Jiaotong University (No. XJTULAC2019‐1272). Chronic stress for 21 consecutive days including food deprivation (24 h), fear sound stimulation (9:00–12:00 a.m. and 15:00–18:00 p.m.), water deprivation (24 h), moist litter (24 h), ice water swimming (5 min, 1‐minute interval, 3 times), empty bottle stimulation (24 h) and day and night inversion (change every 12 h) in turns. All treatment types are listed in Table S1. The control group received no treatments.

### 2.2 Sample collection

Rats were sacrificed after anaesthesia, and then, the testis and epididymis on both sides were separated on ice. Collected tissues were immediately frozen in liquid nitrogen and then transferred to −80°C for storage. The weights of the body, testis, and epididymis were recorded at 3 days, 7 days, 14 days, and 21 days.

Weight gain rate (%) = ((body weight – 0 days body weight) / 0 days body weight) × 100%.

Organ index (%) = (organ weight mg / body weight) × 100%.

### 2.3 ELISA

The concentration of testosterone in the serum was detected with double‐antibody sandwich enzyme‐linked immunosorbent assay (ELISA) kits (Elisa Biotech). Standards and samples were added to the wells, the target antibody and HRP‐conjugated secondary antibody were then added according to the instructions. The data were measured at 450 nm with a microtitre plate reader (FLUO star Omega, BMG LABTECH GmbH).

### 2.4 Tissue protein preparation

Tissue samples were washed with PBS buffer and ground in liquid nitrogen, then dissolved in lysis solution consisting of 7 M urea, 2 M thiourea, 2% w/v CHAPS, 1% DTT and 1 mM PMSF. After ultrasonication on ice (30 s, 0.5 s intervals repeated five times), the samples were incubated on ice (1 h) and centrifuged (10,000 g, 4°C for 30 min). The supernatants were collected; the protein concentrations were determined; and the samples were stored at −80°C.^13^

### 2.5 Two‐dimensional electrophoresis

Samples were diluted in 170 μl (0.8 mg) with buffer (8 M urea, 2 M thiourea, 0.5% CHAPS, 0.02% bromophenol blue and 1% DTT, 0.52% v/v), and applied on immobilized 17 cm, pH 3–10 linear gradient strips. After isoelectric focusing, samples were separated on a 12% SDS polyacrylamide gel and sealed with 0.5% agar. When the bromophenol blue indicator reached the bottom, electrophoresis was stopped, and the gel was stained. Finally, we performed a transmission scan at 300 DPI. Differential protein spots were analysed, and in gel trypsin digestion was performed for further identification.

### 2.6 Mass spectrometric analysis and protein identification

Differential spots in 2‐DE gels were manually excised and washed, and then 50 mM NH_4_CO_3_/CH_3_CN (1:1) was added for decolourization. After vacuum‐drying, 10 mM DTT was added to each sample and reacted at 56°C for 1 h, and samples were then digested with trypsin and NH_4_HCO_3_, washed and dehydrated. The reaction was stopped with 0.1% trifluoroacetic acid, and the solution was collected after centrifugation. After air‐drying, samples and the solution containing 4 mg/ml α‐cyano‐4‐hydroxycinnamic acid matrix in 50% acetonitrile and 0.1% TFA were added in equal amounts. The mass spectra were acquired for peptide mass fingerprinting with an MALDI‐TOF MAS spectrometer with the FlexControl method (Bruker Daltonics). Peptide sequencing was performed with UPLC‐ESI‐Q‐TOF‐MS (Waters Corporation). Instrument parameters and analysis were as previously described.^14^

### 2.7 Immunohistochemistry

Paraffin sections (5 µm) of the testis were deparaffinized and rehydrated with xylene and gradient ethanol (100%, 95%, 90% and 80%), and endogenous peroxidase activity was eliminated. Antibodies were incubated at 4°C overnight after antigen retrieval, and the secondary antibody was incubated the next day. Immunolabelling was revealed with 3,3´‐diaminobenzidine (ZSGB‐BIO). Sections were counterstained with haematoxylin, dehydrated in graded ethanol (80%, 90%, 95% and 100%), rendered transparent with xylene twice and sealed with neutral gum for further analysis.

### 2.8 siRNA synthesis and Transfection

Sequences of Atp5a1 siRNA and scrambled siRNA (Table S2) were pre‐designed and synthesized by the GenePharma Corporation. JetPRIME Transfection Reagent (Polyplus Transfection) was used for the transfection of TM3 cells.

### 2.9 qRT‐PCR analysis

Total RNA was extracted from TM3 cells and then quantified with a NanoDrop Microvolume Spectrophotometer (Thermo). qRT‐PCR was performed with an FTC‐3000P Real‐Time Quantitative System (Funglyn Biotech), with PrimeScript RT Reagent and an SYBR Premix Kits (Genestar). All primers used are presented in Table S3.

### 2.10 MTT assays

TM3 cells were transferred with Atp5a1 siRNAs and cultured at 1500 cells per well in 96‐well plates (five replicates). For the estimation of viable cell number, MTT solution was added to cells and incubated for 4 h and 37°C, and testing followed conventional methods. The cell viability was estimated with an FLUO star Omega plate reader (BMG LABTECH GmbH) at 492 nm.

### 2.11 Transmission electron microscopy

Samples were fixed with glutaraldehyde for 48 h, then dehydrated with an acetone gradient and embedded and cut into semi‐thin sections (0.1 mm). After staining with uranyl acetate and lead citrate, samples were finally observed with a H‐7650 electron microscope (Hitachi).

### 2.12 Western blot analysis

Cells were lysed in RIPA buffer and then centrifuged (800 g, 10 min, at 4°C). Protein quantification was detected with a BCA Protein Assay Kit (Pioneer Biotechnology). Equal amounts of protein (20 μg) were separated with 10% SDS‐PAGE and subsequently transferred to PVDF membranes (Millipore). Membranes were blocked 1 h at room temperature, then incubated with primary antibodies overnight at 4°C (Table S4). The corresponding secondary antibodies were incubated for 2 h at room temperature after TBST washing. Immunoblots were visualized with ECL (Pierce) for chemiluminescence detection.

### 2.13 Data analysis

A Bio‐Rad GS800 scanner was used for two‐dimensional electrophoresis gel scanning. PDQuest professional software was used for qualitative and quantitative mapping analysis for the differential spots from testis tissue proteins in the stressed group and control group.

Protein spots that differed by at least twofold, according to software analysis, were considered differential protein spots and subjected to digestion by trypsin followed by MALDI‐TOF‐MS mass spectrometry. Mass spectrometry analysis was performed with the GPS Explorer software database to determine differential protein‐related information.

qRT‐PCR data were analysed with the 2^−ΔΔCt^ method, and the target gene expression was normalized to GAPDH expression. The positive intensity of immunohistochemistry was scored with H‐score in Image J 2.0 (NIH). All data are expressed as mean ±SD and were analysed in GraphPad Prism version 5.01 (GraphPad Software). *P* < 0.05 was considered statistically significant (*).

## 3 RESULTS

### 3.1 Chronic stress decreases testosterone levels and damages reproductive organs

**FIGURE 2:**
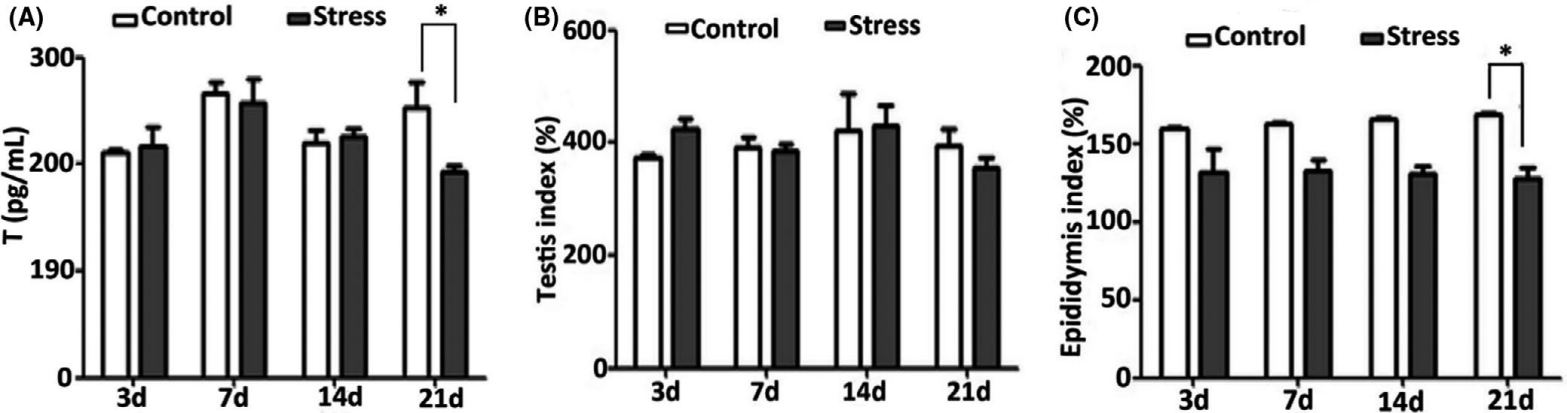
Chronic stress damages male reproductive organs and perturbs hormone levels. A, The concentration of testosterone (T) in the serum in male rats (*n* = 6 per group, **p < 0.01). B, C, Weight index comparison of the testis and epididymis between the stressed and control group (*p < 0.05)

Male rats showed raised hair, nervousness, irritability and mutual fighting on the first day under stress conditions, and showed reduced activity, irregular hair, sluggishness and unresponsiveness after 3 days of stress. Serum testosterone levels significantly decreased after 21 days of stress (Figure 2A). The epididymal index in the stressed group was significantly lower than that in the control group on the 21st day, whereas there was no difference in the testicular index (Figure 2B,C). These results suggested the effects of chronic stress on testosterone levels and reproductive organ damage.

### 3.2 Chronic stress affects testis protein expression patterns

**FIGURE 3:**
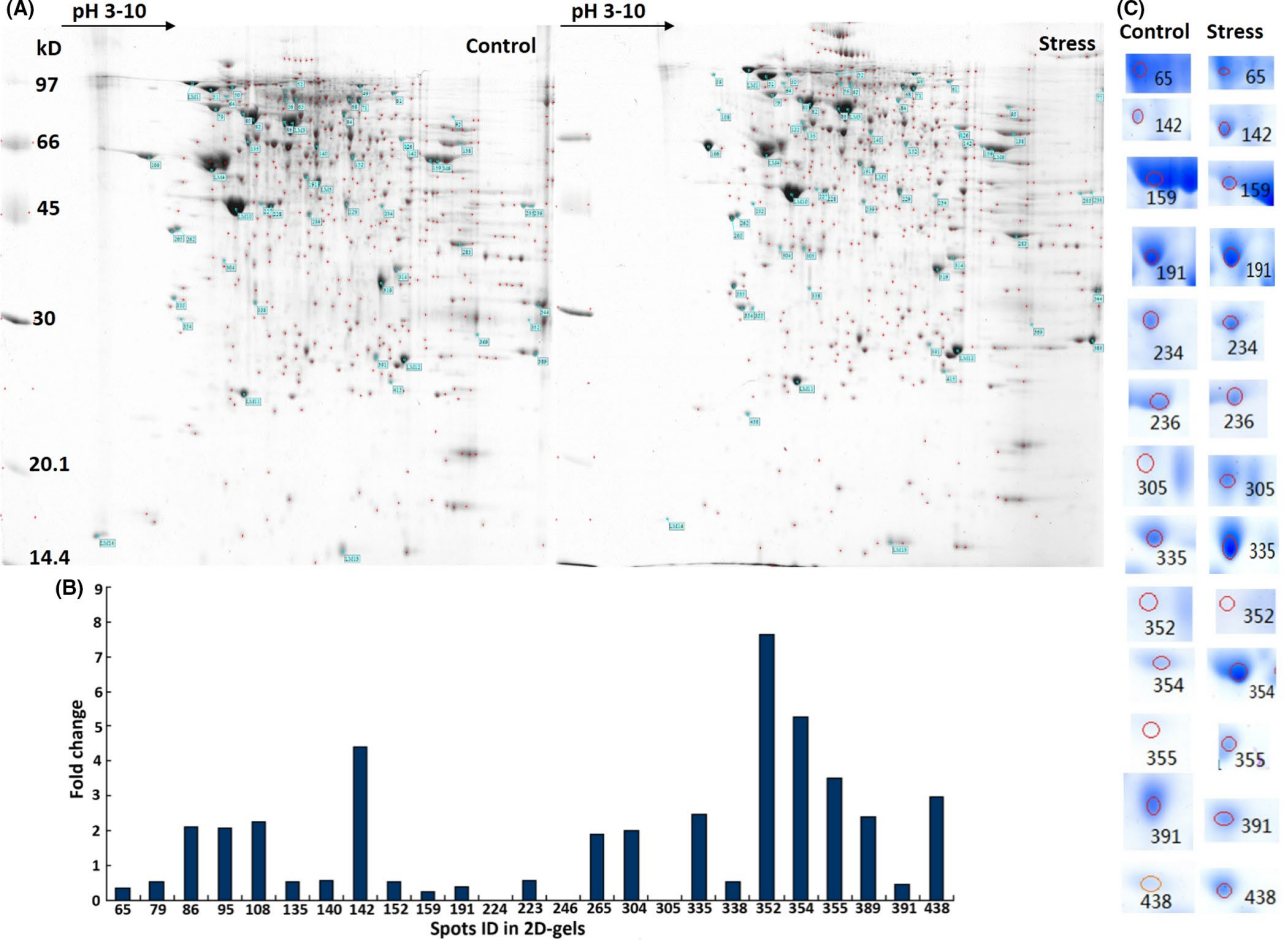
Chronic stress induces differential protein expression in 2‐DE maps of the testis. A, High‐resolution 2‐DE maps of proteins extracted from the testis in rats in the stressed and control groups on day 21 (*n* = 6 per group). B, The replicable protein spots were analysed in PDQuest. A total of 25 differentially expressed spots were screened on the basis of fold differences in gray value relative to the control group. C, Subsequently, 13 of the 25 spots were selected as the most significant in the statistical analysis from the 2‐DE gels. Each spot differentially present between the stressed and control groups is listed. Gels were stained with Coomassie brilliant blue, and the original maps are provided in Figure S1

To assess the effects of chronic stress on the expression of proteins in the testis, we constructed 2‐DE maps with high resolution for the testis at day 21, which showed that 375.5 ± 15.5 and 407.5 ± 17.5 protein spots (mean ± SD) differentiated the control and stressed groups (Figure 3A). The protein profiles were subjected to PDQuest gel analysis, and the results indicated in 25 differentially expressed protein spots (Figure 3B). The 13 spots with the most significant difference were separated from 2‐DE gels, and 10 proteins were finally identified by MALDI‐TOF‐MS (Figure 3C, Table 1, Figure S1). Among these proteins, Akap4, Atp5a1 and Eno1 were downregulated in the stressed group compared with the control group, whereas Pkm2, Prss2, Ywhaz, Acsm2 and Myl9 were upregulated. Got1 and Uqcrc2 were expressed in the stressed group but not the control group. The spectra and molecular weights of these differentially expressed proteins are provided in Table 1.

**TABLE 1:** Mass spectrometry information of differential expression proteins

Spots no.	Gene name	Protein ID	PI	MW	Expression on ratio (S/C)
159	Atp5a1	P15999	8.4	94	0.2:1
191	Eno1	P04764	6.72	86	0.4:1
354	Ywhaz	P63102	5.06	45	5.3:1
142	Pkm2	P11980	8.07	97	4.4:1
246	Uqcrc2	P32551	5.51	67	0.04:*
305/355	Acsm2	O70490	5.18	45	3.5:1
438	Myl9	Q64122	5.14	26	2.9:1
335/352/391	Prss2	P00763	4.99	49	2.5:1
234	Got1	P13221	*	*	0.04:*
65	Akap4	O35774	6.58	97	0.3:1

Note

‘*’ indicates no signal is detected.

### 3.3 Differential protein identification and validation

**FIGURE 4:**
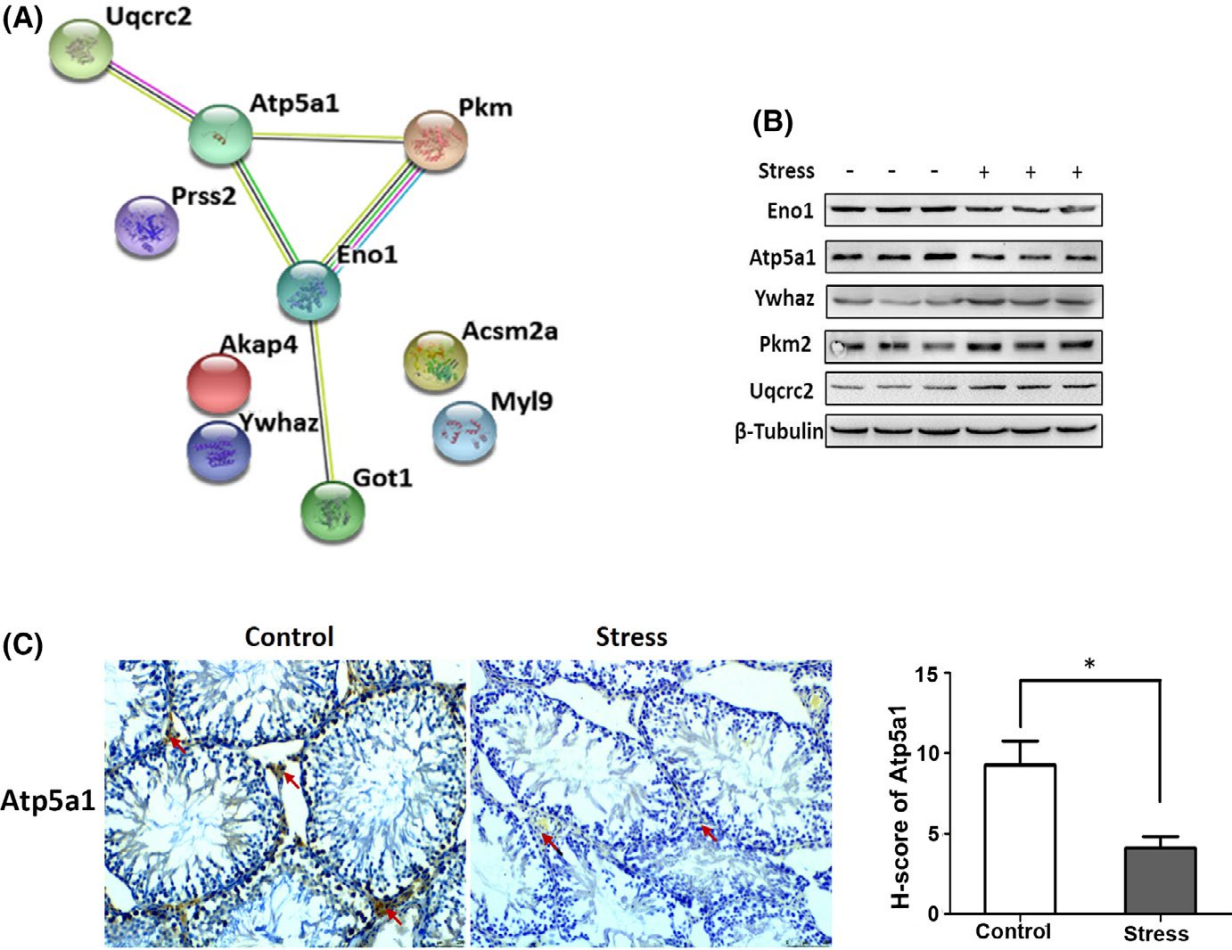
Identification and verification of proteins differentially present in the testes, as induced by chronic stress. A, The 13 most differentially expressed spots were identified as 10 proteins by MALDI‐TOF‐MS. A functional protein association network constructed by STRING showed a strong protein interaction network centred on Atp5a1. B, Western blot analysis of Atp5a1, Eno1, Pkm2, Ywhaz and Uqcrc2 in testis tissue on day 21 showed results consistent with the proteomics findings (*n* = 3 per group). C, The positive expression area of Atp5a1 was mainly distributed in the Leydig cells of testis tissue on day 21 (red arrow, bar = 50 µm), and the H‐score demonstrated a significant difference in the stressed group versus the control group (*p* < 0.05, *n* = 6 per group)

A functional protein association network of the 10 identified proteins was constructed with STRING. These proteins are involved in the production of energy, transcription, protein synthesis, transport, folding and transformation, cell division, apoptosis and oxidative stress, signal transduction, cytoskeleton, flagella and cell motility, cell recognition and metabolism. (Figure 4A). A strong molecular interaction was observed among Atp5a1, Eno1, Pkm2 and Uqcrc2, which are closely associated with mitochondrial energy metabolism. In agreement with the proteomics results, western blot analysis showed that the expression of Atp5a1 and Eno1 was downregulated, whereas that of Pkm2, Ywhaz and Uqcrc2 was upregulated in the stressed group compared with the control group (Figure 4B). In addition, the immunohistochemistry analysis of 21‐day testicular tissue showed that the Atp5a1‐positive region was mainly distributed in Leydig cells, and its expression was lower in the stressed group than the control group (Figure 4C). Overall, these results indicated that the decrease in Atp5a1 was associated with the synthesis of testosterone in Leydig cells.

### 3.4 Atp5a1 affects testosterone synthesis via mitochondrial dysfunction

**FIGURE 5:**
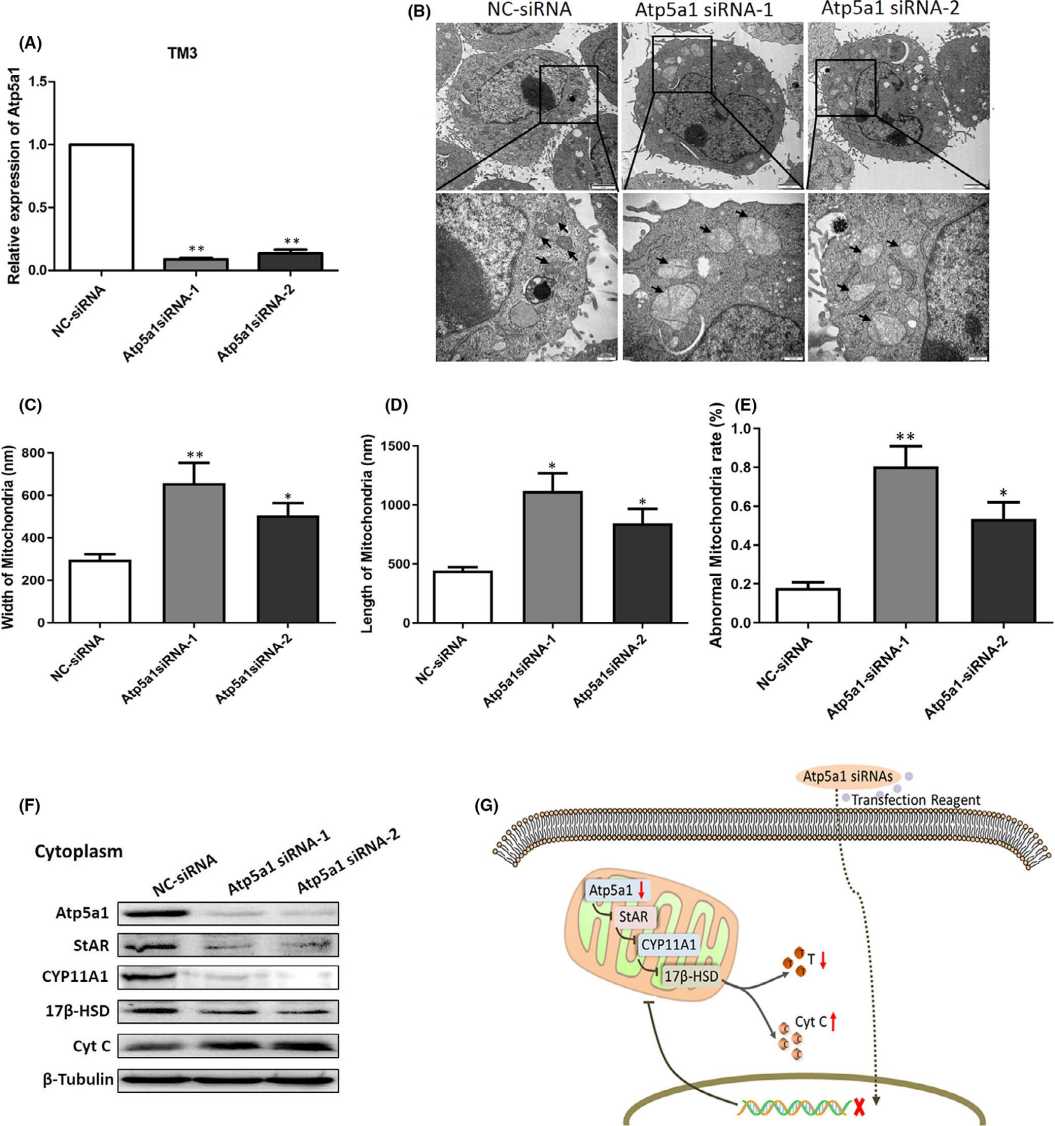
Atp5a1 is involved in the regulation of testosterone synthesis. A, Transfection of TM3 cells with Atp5a1 siRNAs decreased the target gene expression level relative to that of a control gene (GAPDH), as assessed by qRT‐PCR. B, Mitochondria (black arrows) in the TM3 cells were observed by transmission electron microscopy (bar = 2 µm, partially enlarged, 500 nm). The rate of abnormal mitochondria was recorded in at least five random sections. The bar chart shows the length (C), width (D) and rate of abnormal mitochondria (E) in TM3 cells. An asterisk represents statistically significant differences (*p < 0.05, **p < 0.01). F, Expression levels of StAR, CYP11A1, 17β‐HSD and Cyt C in cytoplasm were evaluated by western blot analysis. G, Diagram showing how Atp5a1 transfection in TM3 cells downregulates Atp5a1 expression in mitochondria and promotes mitochondrial damage, thus inhibiting the expression of testosterone synthesis genes

Atp5a1 has been reported to be associated with male sperm motility. Because the immunohistochemistry results revealed that different expression levels of Atp5a1 were mainly distributed in Leydig cells, we speculated that Atp5a1 might be associated with the secretion of testosterone synthesized by Leydig cells. To further clarify this effect, we transfected TM3 cells with siRNAs targeting Atp5a1 in vitro, and the qRT‐PCR analysis showed efficient interference (Figure 5A). Transmission electron microscopy was used to observe the mitochondrial ultrastructure changes in TM3 cells. Many mitochondrial cristae were observed to be tightly arranged, and the inner mitochondrial membrane was intact in the negative control group. However, after siRNAs against Atp5a1 were transfected into cells, the mitochondrial shapes became more heterogeneous, the mitochondrial cristae were clearly broken and arranged unevenly and the inner mitochondrial membrane was incomplete and swollen (Figure 5B). The morphological data statistics showed clearly abnormal length and width, as well as an increased proportion of abnormal mitochondria (Figure 5C‐E). The expression levels of StAR, CYP11A1 and 17β‐HSD distributed in mitochondria associated with testosterone synthesis significantly decreased after Atp5a1 transfection. In addition, the level of cytochrome C (Cyt C) released by mitochondria into the cytoplasm significantly increased (Figure 5F). These results indicated that Atp5a1 restrains the synthesis of testosterone in Leydig cells and may also regulate apoptosis pathway (Figure 5G).

## 4 DISCUSSION

Psychological stress is an important factor for the development of male reproductive dysfunction.^3^ Stress events increase the psychological burden and accelerate illness, including male infertility.^15^ Psychological stress induces depression as well as neurological and reproductive endocrine hormone disorders. Elevated serum corticosterone activates glucocorticoid receptor signalling in the testes, arrests cell cycle progression in spermatogonia and damages male reproductive function. Stress leads to significant decreases in testosterone levels and increases in apoptotic germ cells, which in turn result in a gradual decrease in proteins associated with sperm development and eventually induce male reproductive damage.^16^, ^17^, ^18^ Severe cases can even affect the growth and development of progeny. Animal studies have also shown that chronic stress influences sexual motivation and damages testicular cells in male rats.^19^

Testosterone is mainly synthesized and secreted by Leydig cells in the testis. Blood cholesterol and acetate participate in testosterone synthesis through the smooth endoplasmic reticulum, mitochondria and microbodies. Mitochondria are the most important organelles in cell energy metabolism and one of the main organelles involved in testosterone biosynthesis. Leydig cells contain a series of metabolic enzymes, such as StAR and CYP11A, which are important mitochondrial proteins regulating the transport and metabolism of the precursor of testosterone cholesterol in mitochondria. As an important target of the environment stimulation organelles, mitochondria are involved in mediating apoptosis, inflammation and oxidative stress, and are associated with testicular damage.^20^, ^21^ Previous studies have shown that oxidative stress induces mitochondrial apoptosis and affects testosterone levels,^22^ but few studies have investigated mitochondrial damage under chronic psychological stress, which inhibits the potential mechanism of testosterone generation. This study revealed that stress modulates testosterone synthesis through mitochondrial injury, which plays an important role in the study of male reproduction. We found that chronic stress induced a significant decrease in testosterone levels, and proteomics technology showed changes in testicular gene expression patterns. The 2‐DE and mass spectrometry used in this study identified 10 stress‐related proteins in the testis in male rats, five of which were validated by western blot analysis: Atp5a1, Eno1, Pkm2, Ywhaz and Uqcrc2. These proteins are potentially involved in the regulation of male reproductive function by stress.

Atp5a1 encodes a subunit of mitochondrial ATP synthase that regulates mitochondrial reactive oxygen species generation and is mainly involved in mitochondrial energy metabolism. Atp5a1 participates in biological process including ATP biosynthesis, lipid metabolism and mitochondrial ATP synthesis coupled proton transport. Atp5a1 acts as the mitochondrial complex V component bound Poly(GR), which participates in age‐dependent neuronal cell loss, microgliosis and DNA damage.^23^ Atp5a1 is disrupted by mitochondrial Calpain‐1, thus contributing to diabetic cardiomyopathy.^24^ Through interaction with apoptosis regulators, it also maintains mitochondrial membrane potential and regulates skeletal muscle, thereby contributing to exercise endurance in mice.^25^ However, few studies have found that the downregulation of Atp5a1 is involved in mitochondrial function and sperm quality.^26^ In this study, proteomic analysis and immunohistochemistry verified that the expression of Atp5a1 in the Leydig cells of male rats in the stressed group was significantly diminished. The ultrastructures of TM3 cells transfected with Atp5a1 siRNA were observed by transmission electron microscopy, which revealed more abnormal mitochondria. Furthermore, western blot analysis showed that StAR, 17β‐HSD and CYP11A1, associated with testosterone synthesis in mitochondria, were also significantly reduced. These results suggested that testosterone synthesis via Atp5a1 is an important approach through which stress affects male reproductive dysfunction.

Eno1 is present in the tail flagella in sperm and is involved in the energy required for glycolytic production of ATP for sperm motility.^27^ This protein catalyses the conversion of phosphoglycerate to phosphoenolpyruvate and therefore is a rate‐limiting enzyme in glycolysis; it has been found to be positively correlated with male fertility.^28^ Indirect immunofluorescence assays have revealed that Eno1 also exists in the sperm head, participates in sperm–zona pellucida glycoprotein recognition and promotes sperm–egg‐specific binding.^29^, ^30^ Eno1 was diminished under stress, according to our proteomics analysis in the testis of adult male rats. Thus, Eno1 plays an important role in stress‐induced reproductive damage. Pkm, a major rate‐limiting enzyme in glycolysis, catalyses the transfer of high‐energy phosphate bonds to pyruvate by ATP for the formation of phosphoenolpyruvate and ADP. A novel role of shikonin has been reported in triggering mitochondrial dysfunction, thereby providing a promising therapeutic approach for the treatment of hepatocellular carcinoma.^31^ Pkm2 activity in the serum is significantly elevated in people with cervical cancer, lymphosarcoma, myeloid leukaemia, Hodgkin's disease or myogenic diseases.^32^, ^33^, ^34^ In recent years, researchers have found that Pkm2 exists in the spermatogenic cells of the adult testis and seminiferous epithelium and is involved in spermatogenesis^35^; it is also found in the main segment of sperm flagella and at the junction of the acrosome region and the head midsection of the sperm.^30^, ^36^ Elevated Pkm2 has been observed in sperm dysfunction in men, thus suggesting that Pkm2 inhibits spermatogenesis. Our study showed that Pkm2 increased in the stressed group and led to reproductive organ damage. However, the mechanism through which Pkm2 affects spermatogenesis is not yet clear and remains to be further studied. Ywhaz specifically binds phosphorylated threonine/threonine peptide and phosphorylates the target protein. The protein 14–3–3 zeta is involved in the regulation of cell cycle, apoptosis, transcription and a variety of signal transduction pathways. High Ywhaz expression may serve as a promising prognostic biomarker predictive of poor prognosis in localized prostate cancer.^37^ Ywhaz has also been reported to be involved in spermatogenesis through regulating PP1γ2.^38^, ^39^ Ywhaz and its binding partners play important role in protein–protein interactions during spermatogenesis.^40^ Ywhaz was found in the testis in male rats under stress by the proteomic analysis in this study, thus providing a research breakthrough in testicular reproduction. Uqcrc2 and Eno1 are significantly correlated with fertility.^28^ Uqcrc2 is a component of the biquinol‐cytochrome reductase complex, which is associated with spermatogenesis,^41^ and has been shown to be a biomarker for clinical varicocele and asthenozoospermic testicular cancer patients associated infertility.^42^ The results of our study showed that Uqcrc2 plays a negative role in spermatogenesis,^43^ but the specific pathway requires further study.

Atp5a1 and Uqcrc2 are mitochondria‐related genes. Eno1 and Pkm2 are glycolytic enzymes,^44^ and Pkm2 accumulation in the cell nucleus has been shown to be decreased by Ywhaz inhibition, thus suggesting a close relationship between them.^45^ Mitochondria are important sites in the glycolysis pathway and play an important role in cell metabolism. These results indicated that mitochondrial function is a key component of the stress response in male reproductive damage.

## 5 CONCLUSIONS

This study showed that psychological stress affects body weight and reproductive organs in adult male rats and alters protein expression patterns in the rat testis. More importantly, decreased Atp5a1 involved in the testosterone synthesis pathway in Leydig cells and is potentially closely associated with damage to male reproductive organs. Strengthening mental health and improving lifestyle to reduce the negative effects from stress are important to maintain normal male fertility. However, the incidence of male infertility has continued to rise in recent years. The main underlying reasons are complex, and the pathogenic mechanism of male reproductive dysfunction requires in‐depth exploration in future studies.

## CONFLICTS OF INTEREST

All authors declare no conflicts of interest regarding this study.

## AUTHOR CONTRIBUTIONS

**Xiaofan Xiong:** Data curation (equal); Formal analysis (equal); Validation (equal); Visualization (equal); Writing – original draft (equal). **Qiuhua Wu:** Data curation (equal); Formal analysis (equal); Investigation (equal); Resources (equal); Validation (equal). **Lingyu Zhang:** Data curation (equal); Investigation (equal); Methodology (equal). **Shanfeng Gao:** Data curation (equal); Software (equal). **Rufeng Li:** Data curation (equal); Software (equal). **Lin Han:** Investigation (equal); Resources (equal). **Meiyang Fan:** Data curation (equal); Resources (equal). **Miaomiao Wang:** Data curation (equal); Investigation (equal). **Liying Liu:** Investigation (equal); Project administration (equal). **Xiaofei Wang:** Formal analysis (equal); Software (equal). **Chunli Zhang:** Methodology (equal); Validation (equal). **Yanlong Xin:** Software (equal); Supervision (equal). **Zongfang Li:** Supervision (equal). **Chen Huang:** Review & editing (equal). **Juan Yang:** Conceptualization (equal); Funding acquisition (equal); Writing – review & editing (equal).

## Supporting information

Supplementary Material

## Data Availability

The data that support the findings of this study are openly available.
