# Correction to Chronic stress inhibits testosterone synthesis in Leydig cells through mitochondrial damage via Atp5a1

**DOI:** 10.1111/jcmm.17829

**Published:** 2023-09-19

1

In Xiaofan Xiong et al,^1^ the spots number of ‘224’ and ‘246’ in Figure 3B mismatch with the spots of ‘234’ and ‘236’ in Figure 3C due to technical error. In addition, the spots name of ‘246’ in Table 1 mismatch with the spots of ‘236’ in Figure 3C. The correct Figure 3 and Table 1 are shown below. The authors confirmed that all results and conclusions of this article remain unchanged.

**FIGURE 3:**
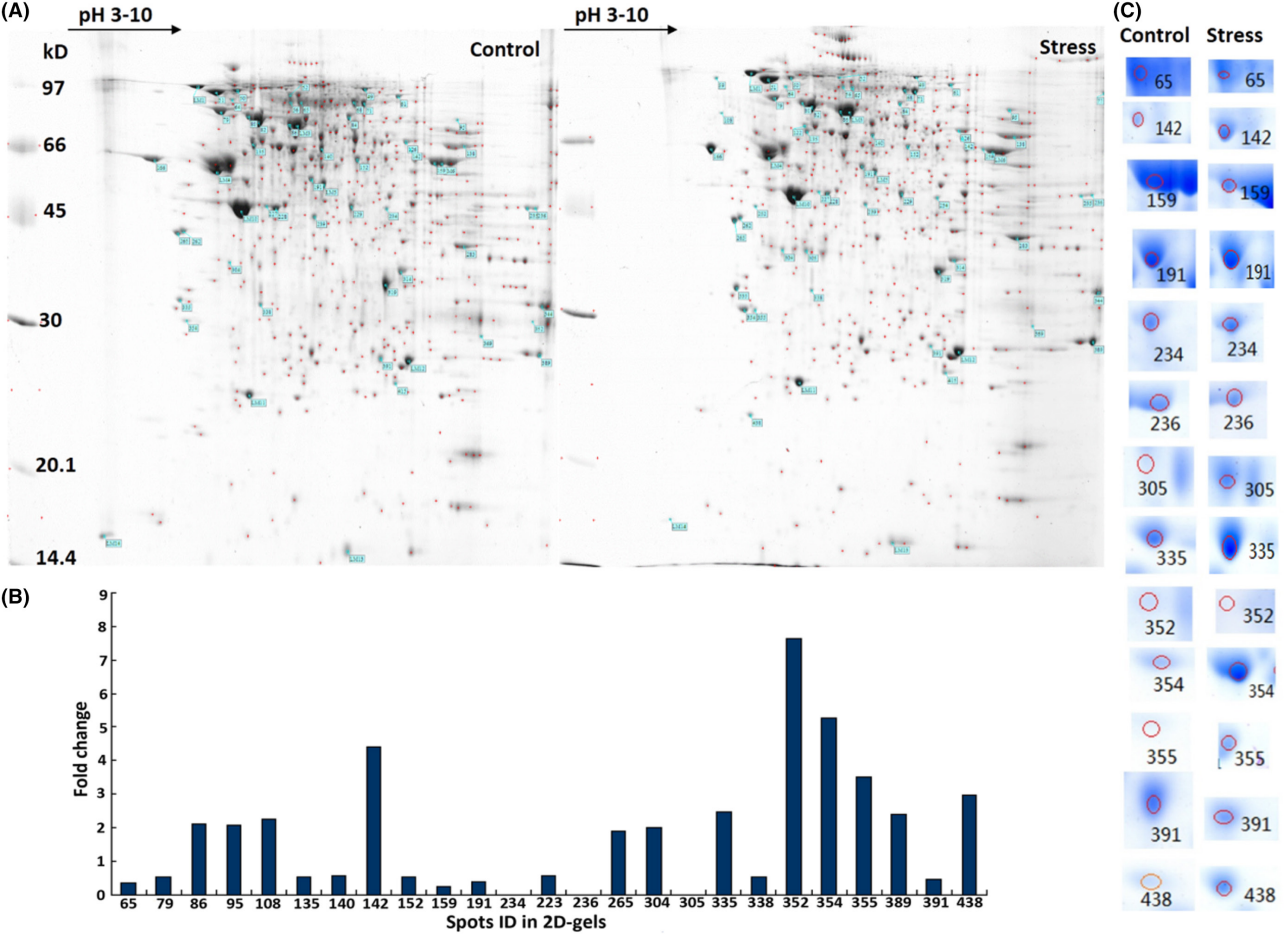
Chronic stress induces differential protein expression in 2‐DE maps of the testis. (A) High‐resolution 2‐DE maps of proteins extracted from the testis in rats in the stressed and control groups on day 21 (*n* = 6 per group). (B) The replicable protein spots were analysed in PDQuest. A total of 25 differentially expressed spots were screened on the basis of fold differences in grey value relative to the control group. (C) Subsequently, 13 of the 25 spots were selected as the most significant in the statistical analysis from the 2‐DE gels. Each spot differentially present between the stressed and control groups is listed. Gels were stained with Coomassie brilliant blue, and the original maps are provided in Figure S1.

**TABLE 1:** Mass spectrometry information of differential expression proteins.

Spots no.	Gene name	Protein ID	PI	MW	Expression on ratio (S/C)
159	Atp5a1	P15999	8.4	94	0.2:1
191	Eno1	P04764	6.72	86	0.4:1
354	Ywhaz	P63102	5.06	45	5.3:1
142	Pkm2	P11980	8.07	97	4.4:1
236	Uqcrc2	P32551	5.51	67	0.04:*
305/355	Acsm2	O70490	5.18	45	3.5:1
438	Myl9	Q64122	5.14	26	2.9:1
335/352/391	Prss2	P00763	4.99	49	2.5:1
234	Got1	P13221	*	*	0.04:*
65	Akap4	O35774	6.58	97	0.3:1

* No signal is detected.
