# Supplementary material for: Chronic stress inhibits testosterone synthesis in Leydig cells through mitochondrial damage via Atp5a1
Source: J Cell Mol Med. 2021 Dec 10;26(2):354–63. doi: 10.1111/jcmm.17085 (PMC8743653; doi:10.1111/jcmm.17085)
Supplement: Supplementary file 1 — Supplementary Material [file JCMM-26-354-s001.docx]

**Chronic stress inhibits testosterone synthesis in Leydig cells through mitochondrial damage via Atp5a1**

Xiaofan Xiong^1,2#^, Qiuhua Wu^3,4#^, Lingyu Zhang^2^, Shanfeng Gao^2^, Rufeng Li^2^, Lin Han^3^, Meiyang Fan^2^, Miaomiao Wang^2^, Liying Liu^3^, Xiaofei Wang^3^, Chunli Zhang^1^,Yanlong Xin^1^, Zongfang Li^1^, Chen Huang^2,3^, Juan Yang^2,3*^

**Figure S1.**

The 2-DE maps stained with Coomassie brilliant blue in control and stress group.

**
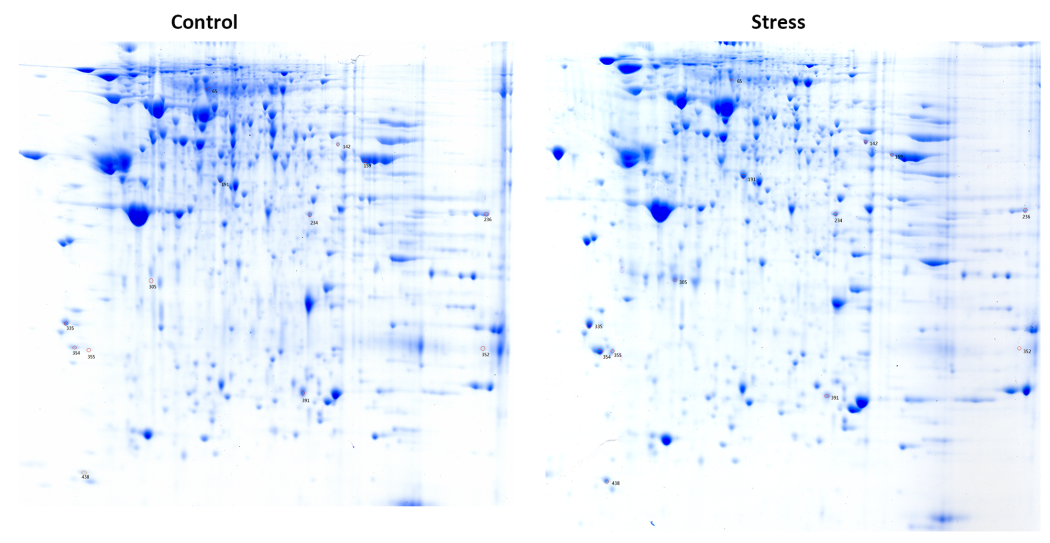
**

**Table S1.**

The stimulations used in chronic stress model.

| Stress type | Operational approach for model group |
| --- | --- |
| Fasting | Forbidden food for 24 h |
| Forbidden water | Forbidden drink for 24 h |
| Empty bottle stimulation | Bottle have no water for 24 h |
| Fear sound stimulation | The sound of fear stimulated for 3 h in a.m. and p.m. |
| Moist litter | Add 200-300 ml of water to the wet the litter for 24 h |
| Ice water swimming | Forced swimming for 5 min into 4 °C water 3 times |
| Day and night inversion | Replacement once after 12 h |

**Table S2.**

Sequences of siRNAs used in this work.

| Name | Sense(5’→3’) | Antisense(5’→3’) |
| --- | --- | --- |
| NC-siRNA | UUCUCCGAACGUGUCACGUTT | ACGUGACACGUUCGGAGAATT |
| Atp5a1 siRNA-1: | GCAGAGGAGAUGGUGGAAU | AUUCCACCAUCUCCUCUGC |
| Atp5a1 siRNA-2: | GCACGCUUUGAUCAUCUAU | AUAGAUGAUCAAAGCGUGC |

**Table S3.**

Primer sequences used for qRT-PCR in this work.

| Gene | Primer sequence | |
| --- | --- | --- |
|  | Forward(5’-3’) | Reverse(5’-3’) |
| Atp5a1 | TCTCCATGCCTCTAACACTCG | CCAGGTCAACAGACGTGTCAG |
| GAPDH | GGTGAAGGTCGGTGTGAACG | CTCGCTCCTGGAAGATGGTG |

**Table S4.**

List of antibodies used in Western blot.

| Gene name | Antibody name | Product number | Brand |
| --- | --- | --- | --- |
| ENO1 | Anti-ENO1 antibody | ab155955 | abcam |
| ATP5a1 | Anti-ATP5A antibody | ab176569 | abcam |
| YWHAZ | Anti-14-3-3 zeta antibody | ab51129 | abcam |
| PKM2 | Anti-Pyruvate Kinase antibody | ab6191 | abcam |
| UQCRC2 | Anti-UQCRC2 antibody | ab14745 | abcam |
| ACTB | β-Actin antibody | sc-47778 | Santa Cruz Biotechnology |
| STAR | STAR Antibody | 12225-1-AP | Proteintech |
| HSD17B1 | HSD17B1 Polyclonal Antibody | A10839 | ABclonal |
| CYP11A1 | CYP11A1 Polyclonal Antibody | A1713 | ABclonal |
| Cytochrome C | Cytochrome C Monoclonal Antibody | 66264-1-Ig | Proteintech |
| β-Tubulin | β-Tubulin Antibody | #2146 | Cell Signaling Technology |
